# Understanding the pathophysiology of *Pseudomonas aeruginosa* colonization as a guide for future treatment for chronic leg ulcers

**DOI:** 10.1093/burnst/tkae083

**Published:** 2025-01-18

**Authors:** Gabriela Gonzalez Matheus, Michelle N Chamoun, Kiarash Khosrotehrani, Yogeesan Sivakumaran, Timothy J Wells

**Affiliations:** Frazer Institute, The University of Queensland, Brisbane, Australia; Department of Dermatology, Princess Alexandra Hospital, Brisbane, Australia; Frazer Institute, The University of Queensland, Brisbane, Australia; Frazer Institute, The University of Queensland, Brisbane, Australia; Department of Dermatology, Princess Alexandra Hospital, Brisbane, Australia; Department of Vascular Surgery, Princess Alexandra Hospital, Brisbane, Australia; Frazer Institute, The University of Queensland, Brisbane, Australia; Australian Infectious Diseases Research Centre, The University of Queensland, Brisbane, Australia

**Keywords:** *Pseudomonas aeruginosa*, Treatment, Chronic wound, Future treatment, Leg ulcers

## Abstract

Chronic leg wounds represent a major burden of disease worldwide, costing health care systems billions of dollars each year. Aside from the financial implications, they also impose a significant physical and psychosocial burden on the patient, their relatives and/or carers, and the community. Whilst measures such as maintenance of wound hygiene, debridement, dressings and compression are the current standard of care, complete healing is not always achievable and ulcer recurrence is common. Thus, there is still a gap to breach in terms of understanding the intricate pathophysiology of chronic wounds and the role this plays on treatment and management. *Pseudomonas aeruginosa* has been linked to poor wound healing, with the pathogen being frequently isolated from chronic leg ulcers. Characterized by its multi-drug resistance, targeting *P. aeruginosa* requires the development of novel therapeutic options. Thus, the aim of this literature review is to describe the pathophysiology of *P. aeruginosa* in chronic leg ulcers and discuss novel treatment strategies. Here, we describe the key molecular mechanisms driving the observed clinical effect of *P. aeruginosa* on wounds and discuss novel strategies of molecular targeting of this common bacteria, establishing new approaches that could benefit patients with chronic hard to heal wounds.

## Background

Chronic leg wounds represent a major healthcare problem, affecting 1%–2% of the population worldwide [1]. They cause significant morbidity, with delayed healing and high rates of recurrence [1]. In chronic ulceration, an inflammatory process leads to an imbalance between pathological factors and the immune defenses promoting the colonization of both Gram-positive and Gram-negative bacteria; with *Staphylococcus aureus* and *P. aeruginosa* representing the most common agents isolated [1].

Chronic bacterial colonization of wounds results in the formation of biofilms, which are polymicrobial populations embedded within extracellular polymeric materials [2]. Biofilms can perpetuate the inflammation cycle within a chronic wound, which in turn delays healing [1,3]. Wounds that are colonized with *P. aeruginosa* are characterized by a greater surface area and tend to heal poorly compared to wounds without its presence [4]. This is compounded by the growing acquired resistance of *P. aeruginosa* to a broad range of antibiotics [1].

In this literature review, we discuss how *P. aeruginosa* affects chronic wound healing, the therapeutic measures currently used to manage *P. aeruginosa* in leg ulcers and novel therapeutic targets to help manage the burden of disease that chronic wounds represent.

## Review

### The pathophysiology of chronic wounds and the role of bacterial growth

Multiple bacterial species colonize chronic ulcers, with 50% of leg wounds usually having between four and six different species [4]. These species include *S. aureus* (93.5% of the ulcers), *Enterococcus faecalis* (71.7%), *P. aeruginosa* (52.2%), *Proteus* species (41.3%), and anaerobic bacteria (39.1%). Once established, many of them persist in the wound [4]. However, the link between the microbial bio-burden in chronic wounds and disease association has not been fully elucidated. This, in part, can be attributed to many studies using solely culture-based approaches which are inadequate in assessing polymicrobial samples [4,5] and are unable to reflect full extent of the diversity of microorganisms present in leg ulcers [6].

*P. aeruginosa* is a Gram-negative opportunistic pathogen that is abundantly present in chronic wounds [7]. Ulcers which are > 10cm^2^ in size and/or have been present for >12 months are more likely to be culture positive for *P. aeruginosa* [8]. Overall, leg ulcers that are colonized with *P. aeruginosa* have a surface area that is three to four times greater than those that are not colonized [4,9]. *P. aeruginosa* can migrate deep into the wound allowing for it to be embedded in the extracellular matrix (ECM) or internalized in keratinocytes in the skin [10]. Once epidermal tissue invasion is established, eradication of *P. aeruginosa* in chronic wounds with antibiotics is highly unlikely [10].

### *P. aeruginosa* virulence factors in chronic wounds

*P. aeruginosa* has the ability to cause direct epithelial damage whilst also impairing epithelial repair mechanisms after injury [10]. This is possible due to an array of virulence factors that the bacteria produces including the flagellum for motility, adhesins for the initial attachment to cells and secretion of various factors that cause damage to the cells and allow the bacteria to persist [11,12]. Many of these virulence factors are regulated by a process known as quorum sensing (QS), which is a cell to-cell signaling pathway that allows the bacterial colony to synchronize their behavior in a way that benefits their survival [13]. QS is vital for biofilm formation and maturation, which in turn also contributes to disease chronicity and antibiotic resistance [14]. All of these factors, combined with the bacteria’s ability to trigger an inflammatory response, promotes delayed wound healing.

*P. aeruginosa* has a single polar flagellum which not only grants it motility, but is also critical for invasion and persistence of the bacteria in sub-cutaneous infection [15]. Additionally, shorter Type IV pili allows the bacteria to attach to host cells and confers twitching motility [12]. Lipopolysaccharides (LPS), a complex glycolipid, are present in the outer membrane of *P. aeruginosa* and contribute to its pathogenicity [10]. LPS is composed of three subunits: endotoxin lipid A, core oligosaccharides and a repeating O-antigen polysaccharide that confers serotype specificity [16]. The most heterogenous portion of LPS, known as the O antigen (or O polysaccharide), has a protective effect, typically conferring resistance to the organism from the killing effect of the host serum [17]. Moreover, the O-antigenic region is highly immunogenic and elicits a high antibody response from the host [17].

In longstanding wounds, the ongoing inflammatory state impacts perfusion, decreases collagen production and causes basement membrane degeneration [18], thus impacting wound healing. The production of exotoxins [19] and cytotoxic substances by *P. aeruginosa* potentiates this process [20,21], and the proteolytic ability of this bacteria means it is able to degrade ECM of host cells, thus contributing to wound chronicity [22]. These toxins are released by Types II (T2SS), III (T3SS), IV (T4SS) and VI (T6SS) secretory systems [10].

T2SS allows for the secretion of elastases (such as LasA and LasB elastases), proteases and enzymes. The T3SS is a needle-like complex used by *P. aeruginosa* to inject bacterial cytotoxins directly into host cells [23]. The main four effectors of T3SS are exotoxin S (ExoS), exotoxin T (ExoT), exotoxin U (ExoU), and exotoxin Y (ExoY) [23], and they have the capacity to alter epithelial integrity whilst hindering repair efficiency [10]. Finally, the T6SS contributes to the competition between adjacent bacterial cells and interactions with host cells [24]. There are more than 30 transcriptional regulators involved in the *P. aeruginosa* virulence network [25], out of which ExsA and CysB have been identified as master regulators [14].

#### *P. aeruginosa* biofilm formation in chronic wounds

*P. aeruginosa* is highly involved in biofilm production in chronic leg wounds [26], with at least 60% of chronic wounds being reported as biofilm containing, compared to only 6% of acute wounds [27]. A biofilm is a structured conglomerate of bacteria and ECM containing polysaccharides, proteins and extracellular DNA [28]. Biofilm formation delays healing [29], protects the bacteria from host defenses [30] and increases bacterial resistance to antimicrobial agents [26].

Biofilm growth and dispersion are promoted by QS, which is used by bacteria to communicate with one another via species-specific signaling molecules [14]. QS uses diffusible small molecules (autoinducers) and allows them to monitor their local population densities [31]. As the population of bacteria grow, these signals reach a specific threshold concentration which promotes alteration of gene expression and initiates a range of behaviors that will benefit the group [31]. These changes include QS regulated virulence factors (such as proteases, elastases, exotoxins and pyocyanin), LPS, flagella, extracellular polysaccharides, and Types II, III, IV, and VI secretion systems [32].

Other factors that may combat *P. aeruginosa* infection such as complement and antimicrobial peptides are also subject to immune evasion during biofilm development [33]. Alkaline protease and elastase produced by *P. aeruginosa* during biofilm growth are able to directly inactivate complement [34]. Moreover, the O-acetylated alginate component found in the ECM of *P. aeruginosa* prevents activation of the alternative pathway of complement, protecting cells from antibody-dependent phagocytosis [35]. Lastly, positively charged antimicrobial peptides can bind to the extracellular DNA component of the biofilm matrix, thus preventing its binding to the bacterial cell surface [36].

#### Immune dysregulation by *P. aeruginosa* in chronic wounds

It has been shown that *P. aeruginosa* has the ability to cause immune system dysregulation, causing an inflammatory response in difficult to heal wounds [5]. Examples of this are increased levels of C3 (a central component in the complement system) [37], increased numbers of neutrophils compared to wounds colonized with other bacteria (such as *S. aureus*) [38] and degradation of the ECM and its components such as cytokines, growth factors and immunoglobulins [39].

A mucoid phenotype has been described for *P. aeruginosa*, and it is characterized by overproduction of the anionic exopolysaccharide alginate [40]. Not only does the overproduction of alginate contribute to biofilm formation, it also inhibits phagocytosis by macrophages and neutrophils, by means of allowing these to accumulate at the surface of the biofilm, thus leaving the activated phagocytic cells in a state of secreting toxic compounds and damaging surrounding host tissues [41]. Notably, the sustained presence of neutrophils throughout the course of chronic infection, as well as the collateral damage caused by these cells is likely another factor in major disease sequelae [42].

### Risk factors for *P. aeruginosa* colonization and infection

All chronic wounds are colonized by microorganisms, however not all colonization leads to clinical infection. In order for pathogens to cause infection, they must be able to survive and replicate in wound tissues by means of developing antibiotic resistance, having the ability to form biofilms and produce virulence factors as well as implement immune evasion strategies [43].

There are various well known risk factors for *P. aeruginosa* colonization and infection, many of these relevant to wound infections. One key risk factor is previous exposure to antibiotics [3,44]. Patients that have been previously treated with antimicrobials, particularly not only aminoglycosides but also quinolones and beta-lactam antimicrobials, are at increased risk of *P. aeruginosa* infection [3]. The underlying reason for this is unclear, and could be due to the selection of antibiotic resistant bacteria by antimicrobial therapy or disruption of the intestinal microbiome composition with changes in the intestinal resistance gene pool [44].

Local factors that can increase the risk for *P. aeruginosa* infection include nutritional availability. Chronic wounds expose subcutaneous tissue and nutrients creating a favorable environment for bacterial growth [43]. In vivo studies have demonstrated that long-chain fatty acids within nutrient-rich wound environments are a major carbon source utilized and are thus crucial in the success of *P. aeruginosa* wound infections [45].

Patient-related risk factors can predispose them to *P. aeruginosa* infection or colonization. Advanced age and immunosuppression—from conditions such as malignancy or after organ transplantation—are linked to increased patient risk of infection with *P. aeruginosa* [3] [44]. Conditions such as diabetes, also predispose patients to *P. aeruginosa* infection. In addition, critically ill patients are also at risk, where concurrent infection and inflammation further predispose the patient to *P. aeruginosa* infection [44]. Environmental exposure is another relevant risk factor as patients who are hospitalized can be exposed to nosocomial strains of *P. aeruginosa* [5], with previous surgery being linked to a higher risk of *P. aeruginosa* infection [3].

Thus, a patient with a chronic leg ulcer is at increased risk of *P. aeruginosa* colonization and infection given they are likely to have been treated in the past with multiple antibiotics (increasing their risk of having drug resistant *P. aeruginosa*), tend to be older in age, are exposed to the hospital inpatient and/or outpatient settings, and likely have underlying conditions that are also immunosuppressive (such as diabetes).

### Current therapeutic measures in chronic lower limb wounds

The management of chronic wounds is quite complex and requires the involvement of a multidisciplinary team that can tailor treatment to each individual patient [46]. Aside from managing the potential underlying cause of the wound (i.e. venous insufficiency, peripheral vascular disease and diabetes.), reducing the biofilm burden is an important element of wound bed preparation [47]. Wound debridement assists in disrupting the biofilm, allowing the wound to progress from an inflammatory state towards healing [48]. Once debridement is performed, cleaning can be done with either normal saline or tap water [49], followed by decontamination with topical antiseptics for colonized wounds [50].

Preparations that are effective against *P. aeruginosa* include octenidine hydrochloride (Octenisept) [50], polyhexanide (Prontosan) [51], and acetic acid 1% [52]. These antiseptic preparations can help reduce the bacterial load and are usually well tolerated. Silver is thought to attach to the bacterial cell membrane and bind key building blocks in the cell [53], hence silver containing products (i.e. silver dressings) are recommended for wounds that are clinically infected or considered at risk of becoming infected with *P. aeruginosa* [54].

Antimicrobial treatment options for *P. aeruginosa* infections tend to be ineffective given the growing prevalence of multidrug-resistant (MDR), extensively drug resistant (XDR), and pan-drug resistant (PDR) *Pseudomonas* strains [44]. Studies have shown that anywhere from 10% to 59% of all bacteria isolated from chronic wounds are MDR strains [55]. Given that *P. aeruginosa* biofilms make the bacteria highly tolerant to antibiotic treatment [56], it is not surprising that *P. aeruginosa* isolates from burn wounds and surgical wounds are more often than not MDR strains [57,58]. This makes therapeutic options limited for the growing number of patients at risk of infection [44], and reinforces the fact that antibiotics should only be used to treat wound that are infected (cellulitis), and not to suppress bacterial colonization to promote wound healing [59].

Despite appropriate medical therapy, leg ulcers tend to recur in 50%–70% of cases [60]. Given that current available therapies to treat *P. aeruginosa* are suboptimal, alternative drugs and new therapeutic options are needed in order to effectively manage and treat wounds infected by *P. aeruginosa.*

### Future therapeutic targets

**Figure 1:**
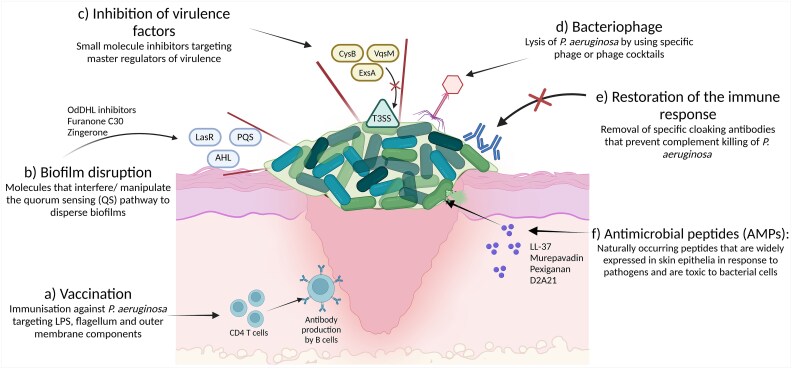
Overview of future therapeutic targets to treat *P. aeruginosa* colonization and/or infection in chronic leg ulcers. (**a**) Vaccinations that lead to production of opsonizing antibodies (**b**) disruption of biofilm formation by targeting QS pathways (LasR and PQS) and signalling molecules (AHL and OdDHL) (**c**) targeting master regulators such as CysB, ExsA, and VqsM to inhibit the T3SS. (**d**) Application of bacteriophage to directly lyse *P. aeruginosa.* (**e**) Removal of cloaking antibodies to allow for complement mediated- killing. (**f**) Delivering AMPs causes bacterial cell lysis. Created in BioRender. Wells, T. (2024) https://BioRender.com/j31j293

Due to the emergence of MDR *P. aeruginosa*, most broad-spectrum antibiotics that are used to treat these infections are now considered ineffective [61], and the creation of new antibiotics is limited by paucity of available targets [14]. Given that antibiotic resistance alone does not explain the virulence of the bacteria, organism characteristics such as the QS system and toxin secretory system may need to be considered in the development of new therapeutic treatments. Novel therapies such as biofilm dispersal agents, antimicrobial peptides, phage therapy, vaccinations and immunotherapy are also being explored as treatment options for *P. aeruginosa* infections (Figure 1). Table 1 summarizes the benefits and barriers identified for each of these therapies.

**Table 1:** Advantages and disadvantages for various of the novel therapies that could be used to treat *P. aeruginosa* infection in chronic wounds.

Therapeutic target	Mechanism of action	Advantages	Disadvantages
Biofilm disruption	Quorum sensing inhibition	● Naturally occurring compounds. ● Can improve wound healing rates. ●Low risk of potentiating antibiotic resistance [62]	● *In vivo* and *in vitro* studies only [63,64]
Antimicrobial peptides	Bacterial cell membrane penetration and pore formation	● Usually have selective toxicity against bacterial cells [65]. ● Some already available for clinical use. ● Use of nanotechnology could help increase safety profile [65]	● High concentrations of free AMP needed for effective antimicrobial activity [65]. ● Risk of local and systemic cytotoxicity with uncontrolled delivery [66]
Virulence factor inhibitors	Blockade of master regulators for Type III and Type VI secretory systems	● New potential drug target to treat *P. aeruginosa* infection. ●Option to control master regulators in order to inhibit infection	● Extensive number of regulators (targets) available [13]. ● Cytotoxic at high concentration in *in vivo* models [14]. ● Further research required in this field
Monoclonal antibodies and vaccines	Specific Immunoglobulins or immunization against OMPs and LPS	● Therapeutic option without risk of causing antibiotic resistance. ● Tested *in vivo* and in clinical trials in patients with lung infections, [67–69], wounds [70] and in humans [71]. ● Can consider developing vaccines against *P. aeruginosa* strains that colonize chronic wounds	● Need to consider creating combinations of antibodies against most of the clinically relevant *P. aeruginosa* serotypes ● Mostly tested in pre-clinical trials only [72]. ●Major focus on lung and bloodstream infections [72,73]. ●None yet available for human use [73]. ● High diversity poses a barrier to development [72]
Restoring immune system	Removal of cloaking antibodies that protect *P. aeruginosa* from serum killing	● Removal of cAbs via plasmapheresis has been used to treat MDR *P. aeruginosa* lung infections [74,75]	● Relevance in chronic wounds yet to be determined
Phage therapy	Bacteriophage lysis of *P. aerugniosa*	● Shown efficacy in *in vitro*, preclinical models and in clinical trials.	● Highly specific for each strain *P. aeruginosa*. ● Resistance can quickly develop. ● Need to optimize delivery

#### Biofilm disruption

Due to the clear importance of biofilm formation to *P. aeruginosa* persistence in chronic wounds, strategies that disrupt or reverse this process could help promote wound healing and reduce the risk of infection. QS has been considered as a target for the development of anti-infective strategies, especially in antibiotic-resistant organisms such as *P. aeruginosa* [62–64,76].

Disrupting biofilms via inhibition of the QS system with either natural or synthetic molecules is a potential option for combating microbial infection [13], and attempts to do so have shown an attenuation of *P. aeruginosa* virulence and biofilm formation in clinical infections [77,78].

*P. aeruginosa* has three major regulatory QS systems including LasI/LasR, Rh1R/Rh1I and *Pseudomonas* quinolone system (PQS)/MvfR [64]. The signaling molecules are known as *N*-acyl homoserine lactones (AHL) and include *N*-(3-oxo-dodecanoyl)-L-homoserine lactone (OdDHL) and *N*-buturyl-L-homoserine lactone (BHL) [62]. LasI produces OdDHL as an autoinducer that is recognized by LasR, and BHL is produced by Rh1I and binds to the Rh1R protein [64]. In the PQS/MvfR system, the PQS affects the MvfR system which is influenced by the expression of both LasR and RhlR [64]. With this in mind, non-native AHLs have been synthesized to act as antagonists and agonists and mimicking the natural autoinducers OdDHL, BHL, and PQS [79,80].

Inhibitors that block QS autoinducers from binding to their respective receptors could thus downregulate the transcription of QS-associated genes and dampen the virulence capacity of *P. aeruginosa* [64]. Park et al. [64] designed a novel pyrone-based QS inhibitor in which the lactone ring of OdDHL was replaced with a pyrone ring and had a long alkyl chain (>9). Using an in silico molecular study, they demonstrated that this compound was able to interfere with the binding of OdHDL to the LasR thus blocking the QS system [64], rendering it a potential treatment option for *P. aeruginosa* infection.

Ginger is known to contain various potent anti-inflammatory compounds including zingerone [63], which has the ability to modulate the biofilm architecture of *P. aeruginosa* [81]. Kumar et al. [63] showed that the use of zingerone *in vitro* reduces *P. aeruginosa’*s amount of hemolysin production as well as elastase and protease activity. It reduces motility phenotypes and significantly suppresses PQS production, which impacts on the biofilm forming capacity due to direct blockade of QS receptor proteins [63]. Validating the use of zingerone as drug that can suppress *P. aeruginosa* biofilm formation in chronic wounds is certainly an option.

Another naturally occurring antagonist, which is an active halogenated furanone, has been isolated from an Australian red seaweed (*Delisea pulchra*) [62]. This compound is known to be a QS inhibitor, and Hentzer et al. [62] used it to developed a novel substance which they termed furanone C-30. In the presence of C-30, *P. aeruginosa* biofilms were efficiently dissolved and became more sensitive to treatment with tobramycin [62]. They showed efficient *P. aeruginosa* clearing with the use of C-30 on *in vivo* models as well [62].

Chemical attenuation of bacterial virulence is an attractive concept mostly because such antipathogenic agents are less likely to pose a selective pressure for development of resistant mutants [62]. Therefore, the use of QS inhibitors might help prevent the formation of detrimental biofilm in chronic wounds, and adding these substances to the patient’s wound care regime could result in quicker wound healing and minimize infection risk.

#### Antimicrobial peptides

Antimicrobial peptides (AMPs) have been researched as therapeutic candidates for the treatment of infectious diseases, including *P. aeruginosa* [82,83]. AMPs are a diverse group of naturally occurring peptides of the innate immune response that are active against pathogens such as bacteria, viruses and fungi, and are produced in large quantities in areas of infection or inflammation [66,84]. A few of the benefits of AMPs is that they rapidly kill bacteria and they are not prone to development of resistance [84,85].

In humans, the two major categories of AMPs are defensins and cathelicidins [86]. These are widely expressed in human epithelia, including the skin, and are created in response to antimicrobial stimuli [87]. Defensins are heavily produced by keratinocytes and are further divided into six types of α defensins (also known as neutrophil peptides) [88] and four classes of human β-defensins (hBD), with hBD 2 and 3 having a significant role in skin protection, being induced by inflammatory (i.e. interleukin-1) and bacterial (i.e. *P. aeruginosa*) stimuli [65]. Moreover, production of hBD 2, 3, and 4 accelerates wound healing via epidermal growth factor receptor (EGFR) stimulated keratinocyte migration [89].

Most cathelicidins have been identified in mammals, with only one being discovered in humans to date [90]. This human cathelicidin (known as hCAP18) is cleaved by serine proteases to release a peptide called LL-37 [90]. This peptide can be found in the epithelia of the skin, and acts as a broad-spectrum antimicrobial by binding and neutralizing LPS and modulating the inflammatory process [91]. Of relevance, LL-37 aids in wound healing by regulating cell migration, inflammation, and angiogenesis [92,93], and in chronic wounds, defects in the production of LL-37 can lead to an active and persistent inflammatory process [94].

There are a range of AMPs which are approved for clinical use as an alternative to antibiotics, including nisin, gramicidin, polymyxins, daptomycin, and melittin [66]. Of these, polymyxins (B and E) show activity against MDR gram-negative bacteria such as *P. aeruginosa* [95], and are considered last line for use in serious systemic infections [96]. For other AMPs with potential clinical relevance to *P. aeruginosa* and chronic wounds, see Table 2.

**Table 2:** AMPs with antimicrobial activity against *P. aeruginosa.*

AMP	Mechanism	Application	Ref
Antimicrobial activity demonstrated against *P. aeruginosa*			
DGL13K	Bacterial cell membrane disruption	Tested in mouse burn wound infection model	[97]
DRGN-1	Antimicrobial and anti-biofilm activity, promotes healing through increased cell migration and proliferation of keratinocytes	Tested in mouse wound infection model	[98]
IRIKIRIK	Bacterial cell membrane disruption	Tested in mouse burn wound infection model	[99]
Novispirin G10	Bacterial cell membrane disruption	Tested in rat burn wound infection model	[100]
Myr-36PW	Bacterial cell membrane disruption and anti-biofilm activity	Tested in mouse wound infection model	[101]
Pseudin-2	Bacterial cell membrane disruption and anti-biofilm activity	Tested in mouse wound infection model	[102]
D2A21 (Demegel)	Bacterial cell membrane disruption	Tested in rat burn wound infection model	[103]
lin-SB056–1	Bacterial cell membrane disruption and anti-biofilm activity	Artificial wound model	[104]
Murepavadin	Inhibition of LPS transporter protein LptD in *P. aeruginosa*	*In vitro* activity against MDR and XDR strains of *P. aeruginosa*	[105]
Hs02	Bacterial cell membrane disruption and anti-biofilm activity	*In vitro P. aeruginosa* biofilm model	[106]
WLBU2	Bacterial cell membrane disruption and anti-biofilm activity	*In vitro P. aeruginosa* biofilm model	[107]
ASP-1/ASP-2		*In vitro P. aeruginosa* biofilm model	[108]
Tested in wound related clinical trials			
Pexiganan	Bacterial cell membrane disruption	Trial completed in adult patients >18yo with diabetic foot ulcers	[109]
LL-37	Bacterial cell membrane disruption	Trial completed in adults >18yo with venous leg ulcers	[110]
PL-5 (Peceleganan)	Bacterial cell membrane disruption	Trial completed in adults >18yo with open wound infections	[111]
PMX-30063 (Brilacidin)	Bacterial cell membrane disruption	Trial completed in adults >18yo with acute bacterial skin infections	[112]
p2TA (AB103)	Immunomodulator that selectively inhibits the binding of superantigen exotoxins to the CD28 receptor on T-helper 1 lymphocytes	Trial completed in adults >18yo with necrotizing soft-tissue infections	[113]
TCP 25	Reduction of LPS-induced inflammatory response	Trial completed in adults >18yo with non-healing leg ulcers	[114]

AMPs show selective toxicity only against bacterial cells. Cationic AMPs do not interact with eukaryotic host cells due to being positively charged and having an amphiphilic nature [65]. However, in practice, high concentrations and repeated administration is needed for free AMPs to have an effective antimicrobial activity, thus leading to loss in selectivity, binding and disruption of mammalian cell membranes [65]. In fact, uncontrolled AMP delivery is associated with local and systemic cytotoxicity [66,115] thus, methods such as nanotechnology are being explored to enhance the stability and efficacy of AMPs and reduce their cytotoxicity [116]. Nanocarrier controlled release systems appear to be promising for sustained and concentration-controlled AMP delivery methods [65]. In order to achieve this, AMPs have to be encapsulated within nanoparticles from biomaterials such as polymeric carriers (i.e. chistosan nanoparticles), inorganic materials (i.e. gold nanoparticles), and lipid-based constituents (i.e. solid lipid nanoparticles) [65].

The AMP LL-37 conjugated with gold nanoparticles has been shown to enhance wound healing *in vivo* compared to the peptide alone, resulting in a higher killing activity against *P. aeruginosa* [117]. Research has also shown that polymyxin loaded solid lipid nanoparticles provide increased occlusive properties, enabling prolonged skin hydration whilst also being effective against resistant strains of *P. aeruginosa* [118]*.* Hydrogel formulations maintain a moist environment and are used in commercial wound dressings [65]. Thus, antimicrobial hydrogels functionalized with AMPs are a potential avenue for managing chronic infected wounds [65]. Polymyxin E, e.g. has been successfully integrated into hydrogels for the treatment of burn wound infections [119].

A synthetic AMP (PXL150) showed a pronounced antibacterial effect against *P. aeruginosa* in vitro, and effectively killed 99.9% of *P. aeruginosa* in *in vivo* models of infected burn wounds in mice in just 4 days of twice daily application, without any signs of toxicity [84].

An alternative would be the use of nanofiber (or electrospun fiber) matrices, and incorporating AMPs (such as LL-37) into these to allow for antimicrobial activity in a controlled release manner [120]. Song et al [121] managed to immobilize a short part of the LL-37 peptide on the surface of silk fibrosin electrospun nanofibers and demonstrated bactericidal activity against *P. aeruginosa*, which was greater at higher fiber density.

#### Disrupting virulence factors

Instead of bactericidal methods, *P. aeruginosa* infection could also be disrupted by targeting the virulence factors themselves. The main targets include the Type III secretion system (T3SS) and master regulators of virulence. T3SS injects toxic effectors into host cells causing acute infection [14], thus interfering with this system has the potential of inhibiting acute infections of *P. aeruginosa*. To date, more than 10 classes of small-molecule inhibitors or antibodies have been developed to inhibit this system [122]. One plausible strategy to disrupt T3SS is by blocking its master regulators [14]. ExsA is a transcriptional regulator that has been identified as a master regulator for T3SS [123], and Grier et al [124] found that N-hydroxybenzimidazole prevents ExsA from binding to the promoters of T3SS genes. Given that *P. aeruginosa* infections result from a combination of multiple genes (rather than a single gene for virulence regulation), controlling the master regulators is an ideal approach [14].

CysB is another regulator that upregulates *P. aeruginosa* pathogenicity by means of increasing swarming, motility, and acts as a positive regulator of ExsA-dependent T3SS transcription [125]. T6SS regulators include—but are not limited to—GacA, AmrZ, and RpoN [126–128], and they control virulence factors such as flagellar motility and biofilm formation [14]. GacA activates T6SS by regulating the expression of the regulatory small RNAs RsmY and RsmZ [129], which function as repressors of the T6SS. RpoN also regulates T6SS by controlling the expression of HcpA and HcpB [130].

In the virulence network for *P. aeruginosa* there are over 30 regulators involved in controlling the QS, T3SS, and T6SS [14]. Examples of these include VqsM [131], VqsR [132], AlgR [133], CdpR [134], RpoN [130], and AnvM [135]. Although no inhibitors have been identified or developed for them, these key virulence-associated transcriptional regulators are potential drug targets for treating *P. aeruginosa* infection [14]. Inhibiting the expression of QS regulators in mice models has proven to reduce *P. aeruginosa* virulence [14], although their high concentration, despite efficiently killing bacteria, can be cytotoxic to mammalian cells [13,136]). There needs to be future studies looking at optimizing these inhibitors to minimize their host toxicity [14].

#### Vaccines and monoclonal antibodies against *P. aeruginosa*

Non-antibiotic treatments such as preventative vaccination and therapeutic monoclonal antibodies (MAbs) are becoming an attractive option to limit and/or manage infection by antibiotic resistance *P. aeruginosa*. Vaccines against *P. aeruginosa* have been in development for many years, however there is yet to be one that is suitable for human use [137]. Of note, most of the efforts have focused on vaccines to prevent lung and bloodstream infections [73], with few studies looking at the development of vaccines to treat infected wounds. Vaccination in *P. aeruginosa* is an extensive topic, hence for an in-detail summary on the potential targets of *P. aeruginosa* vaccination, please see the article by Sainz-Mejias et al (2020) [72].

Relevant to wounds, a trivalent vaccine combining the V-antigen (PcrV), the outer membrane protein I (OprI) and the hemolysin co-regulated protein 1 (Hcp1) tested in murine models showed increased survival rates against *P. aeruginosa* burn wound infections [70]. Another vaccine targeting the flagellin and pilin proteins of *P. aeruginosa* was tested in a burn wound sepsis mouse model, demonstrating the ability of inhibiting systemic dissemination of *P. aeruginosa* infection [138]. Moreover, a vaccine with OMP extracted from four *P. aeruginosa* strains (CFC-101) was successfully used in adult patients with burns >10% total body surface. The vaccine was shown to be safe and highly immunogenic [71]. Thus, there is room for the potential development of vaccines against *P. aeruginosa* strains that colonize and infect chronic wounds.

Passive immunotherapy against *P. aeruginosa* in the way of MAbs represents an alternative or complement to antibiotic therapy, and has shown resolution of infections, shorter hospital stays, and reduced morbidity and mortality [139]. MAbs are generally derived from mice, however a technique to use human B lymphocytes for producing therapeutic MAbs has been developed [140]. MAbs aimed at neutralizing *P. aeruginosa* virulence factors including the PcrV protein and LPS have been created [141].

One of the targets that has been used is the PcrV protein located at the tip of the T3SS injectosome complex [142], with a MAb (V2L2MD) developed by Warrener et al [67] showing high efficacy against *P. aeruginosa* infection in animal models. Topical solutions containing MAbs for treatment of chronic wounds colonized with *P. aeruginosa* can therefore be another therapeutic avenue.

MAbs targeting LPS have also been developed in the form of IgM antibodies [141]. IgMs bind antigen with high avidity and are very effective compliment activators [143]. Panobacumab was developed as an IgM/k isotype directed against the LPS O-polysaccharide moiety of *P. aeruginosa* serotype O11 and was successfully used to treat patients in intensive care with *P. aeruginosa* ventilator associated pneumonia [68]. Clinical trials with egg yolk immunoglobulins (Ig-Y) as passive immunotherapy have also been carried out [69], and the use of an anti-*P. aeruginosa* Ig-Y gargle solution in patients with CF lead to a later time of acquisition of a *P. aeruginosa* positive culture, and later onset of chronic infection [69].

#### Restoring immune system response

Chronic wound fluid (exudate) is vital for healing, and consists of many factors that can resist infection, including high levels of complement proteins [144]. The human complement system is made up of an array of distinct proteins found in serum, and they can react with one another to opsonize pathogens and initiate a series of inflammatory responses that help fight infection [145]. The first component of the pathway—known as C1q—links the humoral immune response to the complement system by binding to antibodies complexed with antigens [145], triggering complement-mediated bacterial killing. This results in the formation of a membrane attack complex (MAC) which creates a pore in the lipid bilayer membrane that destroys membrane integrity, thus killing the pathogen [145].

Bacterial-specific antibody is thus a key component of complement activation. However, in some cases, specific antibodies can have the opposite effect where it protects bacteria from complement-killing. This antibody-mediated impaired complement-killing has been seen in patients infected with gram-negative bacteria such as *Escherichia coli, Salmonella enterica* and *P. aeruginosa* [146,147]. This has more recently been attributed to the presence of ‘cloaking antibodies’ in the form of IgG2, specific for the O-antigen of the bacterial pathogen [148–150]. It is thought that the antibodies cloak the surface of the bacteria in high titers and high affinity, blocking access to the host’s membrane-attack complex (MAC) to the bacterial membrane [151], hence inhibiting complement-mediated serum killing.

Resistance of *P. aeruginosa* to serum killing is considered an important virulence trait [152] and serum-resistant *P. aeruginosa* are commonly isolated from wounds [153]. Despite this, almost 50% of wound isolates still remain sensitive to healthy control serum [152]. How these isolates survive in the complement-rich wound environment is currently unknown.

The presence and impact of cloaking antibodies (cAbs) has been extensively studied in the context of *P. aeruginosa* chronic lung disease. Cloaking antibodies are present in 20% of adult patients with non-CF bronchiectasis [150], 32% of patients with CF [151] and up to 40% in patients post lung-transplant [154]. Importantly, presence of cAbs also associates with worse outcomes in these patients, including worse lung function [150] and higher incidence of chronic lung allograft dysfunction and mortality [151]. These antibodies are also prevalent in acute infections with 24% of patients with *E. coli* urosepsis [149], and 33% of patients with *P. aeruginosa* bacteremia having cAB [155]. In both reports, the presence of cAbs is associated with otherwise serum-sensitive isolates, suggesting cAbs are allowing these bacteria to survive in the blood. Importantly, the prevalence and impact of cAbs in chronic wounds is yet to be elicited.

Finally, removal of cAbs has been used as a novel treatment for multi-drug resistant *P. aeruginosa* infections. In multiple patients with chronic *P. aeruginosa* respiratory infections and cAbs, plasmapheresis was used to remove all antibody from the patient, with donor IVIg given as a replacement [74,75]. These patients became *P. aeruginosa* negative following treatment and thus presents a novel treatment strategy for MDR bacteria. As these antibodies would only be needed to be removed locally in the wound, this may be an attractive option for treatment of *P. aeruginosa* infections of chronic wounds.

#### Phage therapy

Bacteriophages are viruses that undergo viral replication from within a bacterium and can be either lytic (lysis the bacterium upon completion of replication) or lysogenic (integrates into the genome and is inherited by daughter cells and becomes lytic after environmental stressors). The former of which is more useful as a therapeutic [156]. The discovery and research into use of phages for their antimicrobial activity started in the 1910’s, however over the decades issues arose with limited host range and efficacy in the body over time, which coincided with emerging use of antibiotics. Nevertheless, eastern European countries continued their efforts into research into phage therapy, and since the emergence of widespread AMR in recent decades, global interest in phage therapy has formed for a variety of infections [156]. There are multiple recent reviews that broadly detail phage therapy, their use in various wounds and skin infection, and routes of delivery [157–159], and a review of *P. aeruginosa* infections more generally and phage therapy [160]. However, here we focus specifically on phage therapy for *P. aeruginosa-*infected chronic wounds.

Many recent studies have investigated phage therapy in chronic wounds across *in vitro*, *in vivo*, and clinical conditions. *In vitro*, phages have been shown to be effective against 74–92.7% of *P. aeruginosa* isolated from diabetic foot ulcers (DFU) [161,162]. This shows the potential of bacteriophage efficacy refinement with optimization and development. Other *in vitro* studies reported the synergistic effect of phage therapy with antibiotics. Tagliaferri et al compared feasibility of phage therapy as a secondary strategy to antiseptics. *P. aeruginosa* colonies with resistance to antiseptics were sub cultured into further antiseptic dilutions with and without commercially-available NP3 phages cocktail. Secondary exposure to phage or polyhexanide + phage dual treatment resulted in reduced survival of *P. aeruginosa* compared to antibiotic alone [163]. In an artificial wound model, *P. aeruginosa* phage EPA1 combined with gentamycin resulted in significantly higher reduction in biofilm mass compared to phage or gentamycin treatment in isolation [164].

Bacteriophages have also been shown to efficacious in various animal models of wounds. In a rat skin infection model purified phage PhPS127 resulted in decreased *P. aeruginosa* bacterial load, and wound sizes that healed to 60% smaller than the non-treated group [165]. Similar benefits were seen in a mouse skin-infection model, however this study used cloned and isolated lysins from NP1 and NP3 bacteriophages in saline. Two of the three *P. aeruginosa* lysins significantly reduced all bacterial load in the study (PAO1 and clinical isolates) [166]. This shows that the lysins themselves may exhibit broad antimicrobial activity which is useful due to wounds having high diversity of polymicrobial infection. Despite these results, the best evidence of efficacy remains quality clinical trials.

A limited number of clinical trials have used phages to target *P. aeruginosa* in chronic wounds. Two studies from the same research group were undertaken on patients with chronic wounds persisting more than 6 weeks using bacteriophages isolated from different water sources. The initial study had nine of 20 patients with *P. aeruginosa* who received treatment with a three-phage cocktail (for *P. aeruginosa*, *S. aureus*, and *E. coli*) via liquid applied to the wound. All participants achieved sterile wounds by Day 13 [167]. The next study used personalized phage therapy of monophage or phage cocktail depending on the infection via a medicated gauze mesh. In this cohort, wounds containing *P. aeruginosa* had the greatest reduction in area over 90 days compared to wounds with *E. coli* and *Klebsiella pneumoniae* [168]. This demonstrates personalized phage therapy may have improved efficacy. There are many other ongoing phage therapy clinical trials, however the only ones targeting *P. aeruginosa* are with burn wounds and pressure injuries [169].

The current results highlight that phage therapy is a promising therapy to treat *P. aeruginosa* chronic wounds, particularly in conjunction with antibiotic or other therapies. However, several therapeutic concerns remain to be addressed and improved upon. First, bacteriophages often have a very limited host range and resistance to a particular phage does quickly develop, as such a phage cocktail may be ideal, particularly for complex wound environments. To ensure effective phage cocktails, guidelines recommend continually updating formulations with new lysogenic phages, with broad host range, and stable lytic activity [170]There are also regulatory concerns with the production of phages and phage cocktails for therapeutic uses which are reviewed well in a previous elsewhere [159].

Delivery systems are also important and should be further optimized for *P. aeruginosa* wound treatments. Thoroughly reviewed in Mohan et al. 2024, appropriate application of phages needs to be considered to ensure appropriate stable delivery of phage, avoid resistance to the phage from prolonged exposure, and to avoid immune clearance of the phage. The review covered a variety of delivery systems including hydrogels, vesicular systems, nanofibers, and other novel systems [157]. As *P. aeruginosa* tends to infect deeper into wounds than other bacteria, future studies will need to focus on appropriate delivery methods to access the full depth of the wound, such as transferosomes that can migrate deeper in the tissues than liposome delivery, and creative solutions such as dissolving microneedles to disrupt the biofilm of the wound in addition to delivering the phage [157,171].

Finally, for any novel treatment such as phage therapy, safety and patient acceptance are essential. A systematic review assessed the safety of various phage trials and found for difficult-to-treat wounds there were no reported adverse events for topical treatments, and only one mild adverse event for an IV administration [172]. And a focus group was conducted for DFU patients about phage therapy, and at the conclusion of this over 85% would accept phage therapy if recommended by their doctor {Macdonald, 2020 #62].

## Conclusions

Eradicating *P. aeruginosa* from chronic wounds is difficult. Current guidelines for medical treatment of wounds rely on managing the underlying cause for the ulcer, regular debridement to assist in mechanically disrupting the biofilm, cleaning with antiseptic solutions and regular dressings. However, leg wounds are slow to heal and once healed, recurrence rates are high. The need to generate new therapies that target *P. aeruginosa* colonization and infection in chronic wounds has led to promising new avenues being explored including the development of QS inhibitors to disrupt biofilm formation, the use of AMPs as antimicrobials to locally disrupt the bacterial cell membrane, the development of a *P. aeruginosa* vaccine as well as the use of immunotherapy in the form of—either localized or systemic—MAbs. The role of ‘cloaking antibodies’ is yet to be elucidated in chronic wound patients, however studies in CF patients with *P. aeruginosa* infection do suggest that, when present, the removal of these cAbs from blood allows for the infection to be treated. The disruption of virulence factors such as T3SS and other master regulators has also been hypothesized, however further research is needed in this area.

## Data Availability

Data were obtained from public domain resources.
